# INDOOR AIR QUALITY: Wood-Burning Stoves Get Help from HEPA Filters

**DOI:** 10.1289/ehp.119-a164a

**Published:** 2011-04

**Authors:** Carol Potera

**Affiliations:** **Carol Potera**, based in Montana, has written for *EHP* since 1996. She also writes for *Microbe*, *Genetic Engineering News*, and the *American Journal of Nursing*

In many regions wood is seen as a cheap, renewable resource, and burning wood to heat homes is prevalent in rural and urban areas of North America and Europe.^1^ A small, preliminary study suggests air purifiers equipped with high-efficiency particle air (HEPA) filters can lower the amount of indoor fine particulate matter (PM_2.5_) and smoke from woodstoves, potentially reducing residents’ risk of cardiovascular disease from exposure to these air pollutants.^2^ “Our study is the first that I’m aware of that has shown any measurable health benefit from HEPA filtration in wood-burning communities in relatively young, healthy people,” says study leader Ryan Allen, an assistant professor of health sciences at Simon Fraser University, Burnaby, British Columbia.

The researchers monitored 45 nonsmoking adults, average age 43 years, living in 25 homes in Smithers, British Columbia, where residential wood burning is common. Air purifiers costing about $150 were placed in the most active room of the house and the bedroom. The air purifiers ran for 7 days with the HEPA filter inserted and another 7 days without. The order of filter and control conditions was randomly selected for each participant, and participants were unaware of filter status.^2^

Levels of PM_2.5_ and levoglucosan, a validated tracer of woodsmoke, were measured inside and outside the homes. At the end of each 7-day period, blood and urine samples were assessed for markers of inflammation and oxidative stress, and microvascular endothelial function was measured by peripheral artery tonometry.^2^

Use of the HEPA filters reduced indoor PM_2.5_ concentrations by 60%, and indoor levoglucosan levels fell by 75% on average, compared with nonuse. HEPA filtration was linked to a 9.4% increase in the reactive hyperemia index (RHI), a marker of endothelial function, and a 32.6% decrease in C-reactive protein, a marker of inflammation.^2^ A reduced RHI reflects an impaired blood vessel response to changes in blood flow and is an early indicator of atherosclerosis.^3^ These physical changes occurred even though PM_2.5_ levels were relatively low to begin with—about 11 μg/m^3^ outdoors compared to the U.S. Environmental Protection Agency’s annual average standard of 15 μg/m^3^.^4^

Even people who don’t use woodstoves themselves may benefit from HEPA filters, Allen says. “Most stoves don’t put smoke into your living room directly,” he explains; instead, smoke that is vented outdoors leaks back into nearby homes through cracks around doors and windows.^2^

A larger, better-controlled study is needed to confirm these findings, as well as determine any long-term health benefits of filtering indoor air, such as preventing strokes or heart attacks. Still, these initial results are promising in a world where indoor air pollution from solid fuels such as wood is a top global risk factor for disease and premature death.^5^ Moreover, says Lars Barregard, a professor of occupational and environmental medicine at the University of Gothenburg, Sweden, “The use of wood for heating may become more common as the cost of other fuels rise or fossil fuels are restricted.”
